# Gait Variability and Complexity during Single and Dual-Task Walking on Different Surfaces in Outdoor Environment

**DOI:** 10.3390/s21144792

**Published:** 2021-07-14

**Authors:** Denisa Nohelova, Lucia Bizovska, Nicolas Vuillerme, Zdenek Svoboda

**Affiliations:** 1 Department of Natural Sciences in Kinanthropology, Faculty of Physical Culture, Palacký University Olomouc, 771 11 Olomouc, Czech Republic; lucia.bizovska@upol.cz (L.B.); zdenek.svoboda@upol.cz (Z.S.); 2 Laboratory AGEIS, Université Grenoble Alpes, AGEIS, 38000 Grenoble, France; nicolas.vuillerme@univ-grenoble-alpes.fr; 3 LabCom Telecom4Health, Orange Labs & Université Grenoble Alpes, CNRS, Inria, Grenoble INP-UGA, 38000 Grenoble, France; 4 Institut Universitaire de France, 75231 Paris, France

**Keywords:** trunk acceleration, sample entropy, root mean square, manual dual-task, cognitive dual-task, daily living gait, real life gait

## Abstract

Nowadays, gait assessment in the real life environment is gaining more attention. Therefore, it is desirable to know how some factors, such as surfaces (natural, artificial) or dual-tasking, influence real life gait pattern. The aim of this study was to assess gait variability and gait complexity during single and dual-task walking on different surfaces in an outdoor environment. Twenty-nine healthy young adults aged 23.31 ± 2.26 years (18 females, 11 males) walked at their preferred walking speed on three different surfaces (asphalt, cobbles, grass) in single-task and in two dual-task conditions (manual task—carrying a cup filled with water, cognitive task—subtracting the number 7). A triaxial inertial sensor attached to the lower trunk was used to record trunk acceleration during gait. From 15 strides, sample entropy (SampEn) as an indicator of gait complexity and root mean square (RMS) as an indicator of gait variability were computed. The findings demonstrate that in an outdoor environment, the surfaces significantly impacted only gait variability, not complexity, and that the tasks affected both gait variability and complexity in young healthy adults.

## 1. Introduction

Recently, studies using a nonlinear approach for gait evaluation highlighted the fact that normal human gait is fundamentally chaotic, highly complex and provides flexibility to adjust to disturbances that occur during movement [1]. In daily life walking, people are exposed to multiple and changing environmental conditions. Urban and country environments contain various surfaces, such as pavers, cobblestones, asphalt, concrete, gravel, grass, sand and soil. It has been previously demonstrated that walking on an uneven surface modifies lower limb and trunk kinematics, spatial−temporal gait characteristics and their variability even in young adults [2,3,4,5,6,7,8,9]. Gates, Wilken, Scott, Sinitski and Dingwell (2012) [5], who compared level ground with a destabilizing loose rock surface, confirmed that the variability of step parameters (length, time, width) increased twofold on the rock surface. Irregular surfaces formed by triangular wooden prisms also affected the step width variability and step time variability and this effect was even more pronounced in older women, compared to young women [7]. Significantly greater also was the step time variability, pelvis acceleration variability (root mean square) and acceleration amplitude variability in all directions while the pelvis harmonic ratios decreased significantly in the anteroposterior (AP) and mediolateral (ML) directions on artificial grass underlain with foam and wooden blocks, compared to a level corridor [6]. In the above mentioned studies [5,6,7], the surface conditions were simulated trying to reach real world conditions and all of them reported changes in the observed variables relating to gait pattern. However, artificial surfaces are not directly comparable to everyday surfaces. Despite the fact that nowadays, gait assessment in real life conditions is possible thanks to the availability of wearable sensors, we found only one study evaluating gait parameters under various real life conditions [10]. This study, focused mainly on 30 diabetic patients and 15 healthy older people as a control group, compared spatial−temporal gait characteristics and gait kinematics on three different surfaces (tar, grass and cobblestones). Alterations in gait pattern of healthy older adults [10] and also in diabetic individuals [10] were observed. Gait characteristics (including stride-to-stride variability) were altered especially during walking on cobblestones in comparison with grass and with tar [10]. 

Furthermore, in everyday life, it is also common to walk and concurrently perform one or more tasks (e.g., talking, carrying something, reading signs, thinking—planning, solving problems) [11]. Since motor and cognitive function share the same neuroanatomical structures and psychological processes [12], multitasking may induce interference effects due to limited processing capacity and central overload [13,14]. Subsequently, this leads to performance (motor, cognitive or both) impairment, which is dependent on the type, the complexity and the difficulty of the tasks (e.g., see [15] and [16] for recent reviews). Some tasks can also influence gait performance in young adults [11,17,18], but the results showed contradictory tendencies in view of the fact that the given tasks and instructions were different. Indeed, gait performance could also be differently influenced by altering the focus of attention [19].

The acceleration signal, obtained from inertial measurement units attached on various body segments, is commonly used in gait analysis and this approach is applicable also in real life gait assessment. The validity of the trunk acceleration measurement to monitor gait during real world situations was previously confirmed by Pizzamiglio, Abdalla, Naeem, and Turner (2018) [20]. The acceleration signal allows the computation of various parameters to evaluate gait (e.g., see [21,22] for recent reviews). The most elementary and most commonly used variable describing the dynamics of gait pattern is root mean square (RMS) of trunk acceleration [23,24]. RMS is an indicator of acceleration data dispersion around zero [24] and indicates the magnitude of acceleration [25]. However, computing the magnitude of variability may not be sufficient to understand the human movement control mechanisms [26]. Therefore, an alternative analysis (i.e., nonlinear methods) derived from the theory of stochastic dynamics, came to the fore, although evaluation of gait variability is still recommended due to the presumed accuracy in identifying subtle changes in walking owing to pathological disorders or illnesses and with the assumption that higher variability indicates reduced consistency and a more unstable gait pattern [27]. Sample entropy (*m*, *r*, N) (SampEn), which describes the complexity of the movement in terms of performance automaticity, is defined as the negative natural logarithm of conditional probability that two sequences that are similar for *m* points remain similar at the next point within a tolerance *r*, where self-matches are left out from the probability calculation [28]. SampEn quantifies the similarity, the regularity and the complexity of the time series and provides indirect information about the stability of performed activity. Higher values of SampEn correspond to a lower similarity of the system which results in higher complexity, in other words, higher values indicate less periodicity, less regularity and more unpredictability [28]. Thus, regularity in the time series could be an indirect indicator of dynamic stability in the human gait [26,29]. Additionally, SampEn has also shown higher reliability for short data sets, which are commonly analysed in gait studies [30] and slightly higher sensitivity to changes in gait dynamics [31]. 

So far, a limited number of studies have used sample entropy to compare single and dual-task walking performance. None of them observed real life gait, but two of them observed gait in a corridor at a self-selected speed [32,33], and another two studies assessed gait on a treadmill at a predefined speed [34,35].

Along these lines, different external conditions (i.e., environment, task) may have altered the demands on gait and posture control, mainly through balance (dynamic stability) and the ability to adjust to the environment [36]. For the maintenance of successful gait performance in demanding conditions, we can expect some adaptation—gait pattern should modify itself, taking into account not only the internal and external influences but also efficiency of movement. However, even if it is essential for humans to be able to walk in various conditions and simultaneously perform the tasks, the effects of the walking conditions and the tasks during outdoor locomotion have not been studied yet in detail. Therefore, the current study presents an original comprehensive gait evaluation, where the various areas of interest are assessed and combined together (e.g., outdoor environment, surfaces, dual-tasks, linear—variability and nonlinear—complexity analysis). The goal of this study was to assess the effects of different dual-tasks while walking in an outdoor environment on different surfaces on gait variability and gait complexity in young healthy adults.

## 2. Materials and Methods

### 2.1. Participants

The research sample consisted of 29 healthy young adults (18 females, 11 males) with the following descriptive characteristics (mean ± standard deviation): age 23.3 ± 2.9 years, height 174.8 ± 9.9 cm and weight 68.0 ± 11.1 kg. The inclusion criterion was the age between 20–30 years. The exclusion criteria included serious injuries and lower limb or spine surgery, neurological, pulmonary, cardiovascular and other diseases or drugs affecting balance or gait, pregnancy, alcohol or drug consumption 12 h prior to measurement and medium or high physical activity 48 h prior to measurement.

### 2.2. Ethics Approval

The study was approved by the institutional ethics committee (the Ethics Committee of the Faculty of Physical Culture, Palacký University Olomouc, Olomouc, Czech Republic, no. 78/2018). All participants were acquainted with the aim of the study and the testing protocol. All participants signed written informed consent, prior to the measurement, containing basic information about research.

### 2.3. Experimental Protocol

Participants wore sports clothes and used their own comfortable sports shoes. They were instructed to walk 45 m at a preferred walking speed as straight as possible, to maintain a stable pace and not to stop during the trial.

Gait assessment was executed in an outdoor environment on three different surfaces (asphalt, cobblestones, grass) with and without concurrent manual or cognitive task (i.e., single-task, dual-task manual, dual-task cognitive). These three outdoor road conditions (asphalt, cobblestones, grass), represent common walking situations encountered in everyday life. On each of this surfaces, the following three different walking situations were executed.

#### 2.3.1. Single-Task—Only Walking

The participants received following instructions: “Now you are going to walk on a grass/cobblestones/asphalt. Walk as usual, in your own natural speed, like you would walk when you would be on a trip. Walk as straight as possible. Do not stop walking and do not speak during the measurement”.

#### 2.3.2. Dual-Task Manual—Carrying a Cup Filled with Water

The participants received the same instructions as during the single-task. Additional information referred to the manual task was as follows: “Try to walk and carry a cup filled with water, do not spill the water”.

#### 2.3.3. Dual-Task Cognitive—Subtracting Number 7

The participants were briefed as during the single-task. Additional information pertaining to the cognitive task was as follows: “Try to walk and loudly subtract number 7 from number 1000/1500/2000. Do these two tasks simultaneously, do not stop walking or counting”. This cognitive arithmetic task involves working memory and thus is linked with executive function [37]. Considering the task repetition by young adults we chose to change the number for subtraction to maintain the difficulty and to avoid learning effect (memory).

For the dual task conditions, no prioritization was given to a single task, participants were asked to perform both tasks as well as possible [38,39].

For each experimental condition, participants performed a single trial, representing a total of 9 trials per subject. The order of presentation of the nine experimental conditions was randomized over participants.

### 2.4. Instrumentation 

Gait speed was computed for each trial from the time needed to walk through middle 40 m of the walkway.

ML, AP and vertical (V) trunk accelerations were recorded during gait with an inertial measurement unit (Physiolog, GaitUp, Lausanne, Switzerland, sampling rate 128 Hz). The unit was attached above the L5 vertebra of the lumbar spine and secured by a double-sided tape on the skin allowing free body movement. Gait was tested under a single task and two dual-task conditions.

### 2.5. Data Analysis/Processing

Raw accelerations were used for further processing. After removing mean, heel strikes were identified based on the algorithm of Zijlstra and Hof (2003) [40]. Fifteen middle gait cycles were used for analysis with computation of a variability indicator—RMS [6]—and complexity indicator—SampEn (*m* = 2; *r* = 0.15)—for each direction of the movement [41]. Data were processed without prior filtering in MatLab (R2019b, MathWorks, Inc., Natick, MA, USA) with SampEn computation scripts available on Physionet [42,43,44].

In total, we computed the undermentioned gait parameters:

- Gait speed
- RMS from trunk acceleration in ML, AP and V direction
- SampEn from trunk acceleration in ML, AP and V direction

### 2.6. Statistical Analysis

The statistical software Statistica 12 (Stat-Soft, Inc., Tulsa, OK, USA) was used for statistical data processing. The normal data distribution of all computed parameters was verified by the Kolmogorov−Smirnov test. Analysis of variance (ANOVA) for repeated measurements was then applied to the data to examine the effect of 3 surfaces (asphalt versus cobblestones versus grass) and 3 tasks (single versus manual versus cognitive) and interaction effect of surface and task on gait.

The level of statistical significance for all tests was set at α = 0.05. Bonferroni post hoc test was performed when significant effect of task or surface was detected.

## 3. Results

The results are presented in Table 1.

The analysis of the gait speed showed no main effect of the task, no main effect of surface and no interaction effects of the surface and task for all directions (V, ML, AP).

The analysis of the RMS of trunk acceleration showed the main effects of the task on RMS in all directions—V (*p* < 0.001), ML (*p* < 0.001) and AP (*p* = 0.004) and the main effects of the surface in the ML (*p* = 0.006) and AP (*p* = 0.002) directions. The effects of surface on RMS in the V direction and the interaction effect of surface and task on RMS in all directions (V, ML, AP) were not found.

The analysis of SampEn of trunk acceleration showed the main effect of task on SampEn in the V direction (*p* = 0.006). No main effect of task on SampEn in the ML and AP directions, no main effect of surface on SampEn in all directions (V, ML, AP) and no interaction effects of surface and task on SampEn in all directions (V, ML, AP) was found.

Furthermore, the Bonferroni post hoc test revealed the significant difference for RMS of the trunk acceleration in ML and AP directions, respectively, in the case of manual (*p* = 0.028; *p* = 0.028) and cognitive tasks (*p* = 0.049; *p* = 0.017) performed on asphalt and grass and then between the manual task and single-task (*p* = 0.010) performed on asphalt (ML direction).

The Bonferroni post hoc test did not reveal a significant difference for trunk acceleration SampEn in the V direction. Non-significant differences were found for trunk acceleration SampEn in the V direction between cognitive and manual tasks performed on asphalt (*p* = 0.786) and grass (*p* = 0.480).

## 4. Discussion

The present study was designed to assess the effects of various conditions (i.e., two types of dual-tasks, three types of surfaces) on gait performance in an outdoor environment in healthy young adults.

Our main results can be summarized as follows: (1) the dual-tasks affected gait performance in young adults in terms of gait variability and complexity; and (2) the surfaces altered gait variability but not complexity in young adults.

### 4.1. Effect of Surfaces on Gait Speed

Gait speed did not differ significantly on the various surfaces, which indicated that young healthy adults were able to maintain their natural gait speed in spite of more demanding conditions. We deliberately chose ordinary surfaces, which are common in the real world environment and we supposed that our participants had previous experience of them. Not so usual surfaces (wet linoleum, soft foam with small wooden blocks covered by artificial grass, triangular wooden prisms underneath one layer of industrial carpet, soft foam) specifically constructed in laboratory settings probably added more challenge to gait control (unknown conditions), therefore in some cases a decreased waking speed in the young adults was found [5,45,46], but not in others [6,7,47,48]. However, this discrepancy could be caused by different experimental protocols (walking velocity calculated from spatial−temporal gait variables or derived from the trunk acceleration). Our selected surfaces obviously did not pose a threat to young healthy adults, so there was no reason for coping strategies or adaptation, involving a decline in gait speed.

### 4.2. Effect of Tasks on Gait Speed

Furthermore, the young adults’ gait speed remained unchanged during the dual-task walking. This finding is in contrast with the others, who reported slower gait speed during walking with secondary cognitive task [46,49,50,51,52,53,54,55] and also manual tasks [53,56]. In all these cases, however, the performances were followed in laboratory settings and with shorter walking distances. As recent studies comparing dual-task walking in laboratory and free-living environments did not find significant effects of the environment on the young adults’ gait [54,55,57], other dissimilarities should explain our results—especially task complexity, attentional distribution and walking distance. Because we assessed over a longer distance and only mean gait speed, the young adults had more time to deal with the task, which was presumably simple for university students, not overloading their capacity. Further, the instructions were different from ours in some cases. We instructed the participants to fulfil both tasks simultaneously, but in other studies the participants were asked to concentrate on secondary task [53] and this shift in attention could support the speed decline more. Indeed, changes in gait speed under dual-tasking are, in young adults, associated with task prioritization. The focus of attention on gait showed a smaller decrease in comparison with cognitive or no priority; and no prioritization and cognitive task prioritization caused a similar speed decline [19]. 

Nevertheless, there are also studies such as ours, which in the case of manual tasks (holding a phone) did not find any changes in the young adults’ gait speed [54].

### 4.3. Effect of Surfaces on Gait Variability

There is a wide range of studies evaluating the effect of surfaces on gait variability, usually in laboratory settings with linear methods of assessment. We assessed gait in an outdoor environment on real world surfaces, which should be closer to usual real life gait. With increasing surface difficulty (even asphalt, bumpy cobbles, uneven grass) trunk acceleration variability in the ML and AP directions tended to increase, the highest values were observed during walking on grass. Grass, as a representative irregular surface, produced more perturbations, which should be processed by the human control system. Surface irregularities were projected in the alteration of ML trunk acceleration variability which is supposed to be closely related to stability [26,58,59,60] and also in changes of AP trunk acceleration variability which represents gait fore−aft dynamics [26,58,59]. Moreover, we suppose that the RMS values were not influenced by different gait speeds as no effect of surfaces and tasks on gait speed was found.

Our findings are in accordance with those reported in previous studies [2,3,4,5,6,7], which reported increased variability in variables evaluating gait in simulated conditions executed in laboratory environment.

When young adults walked on uneven surfaces, which provoked higher demands on their stability and ability to adjust to the environment, they adopted a more variable gait pattern, which could be considered as “unstable”. Given the circumstances, it is rather a “flexible-adapted” gait pattern in unstable conditions. Thus, we confirmed that young adults gait variability increases with surface irregularities, even in an outdoor environment.

### 4.4. Effect of Tasks on Gait Variability

Although a wide range of studies focusing on dual-task walking exist, only a few studies focus on the evaluation of the effect of the tasks on gait variability in young adults. Our results showed that a secondary task also altered the young adults’ gait variability, when the variability of trunk acceleration in all directions (V, ML, AP) tended to decrease during performing manual and cognitive secondary tasks, even when walking on different surfaces. The exception was the cognitive task, which was performed on grass, where the variability of trunk acceleration in all directions tended to rise. We expected that the combination of grass and a secondary task would pose the most challenging condition.

In some studies, a decreased variability of the young adults’ spatial−temporal gait parameters (minimum toe clearance variability, toe clearance in swing, centre of mass height in stance) during the cognitive dual-task (subtraction) walking was reported [61,62], although Hamacher, Hamacher et al. (2019) [62] simultaneously found significantly increased stride time variability and unchanged stride length variability. However, in contrast with these results, Laessoe et al. (2008) [53] observed no significant changes in young adults’ gait variability (temporal stride to stride variability, trunk acceleration stride variability) during the performance of manual (holding sticks) and cognitive (subtractions of seven) tasks, compared to single-task walking, and Santhiranayagam et al. (2015) [56] during the performance of a manual task (carrying a glass of water) compared to a single task (gait variability of minimum toe clearance). It follows that various tasks may alter different domains of gait pattern in a different manner.

In some cases, “improvements” (lower variability) in motor performance of skilled movements during the inclusion of a secondary cognitive task have been shown and explained due to the shift in focus of attention away from movement control which in turn leads to movement performance improvements [63,64]. Reduced gait variability in the dual-task conditions could be caused by reduced (sensorimotor) noise as was shown by Hamacher, Koch, et al. (2019) [61]. Carrying things belongs to the activities of daily living, therefore we could consider this action as a learned skill, which is controlled at the level of the subcortical brain centres (cerebellum, vestibular system, etc.). Carrying things in a specified position (cup filled with water) during walking certainly impacts movement dynamics, through different postural adjustment, upper limb restriction and also, as our results revealed, through trunk movement restriction. To complete this manual task (i.e., to keep the upper limb in a “steady” horizontal position with as little deflection as possible), an appropriate trunk “stiffness” is required and this stiffness manifests itself with a decrease in movement variability. Although this gait pattern could be described as “more stable”, decreased variability could also mean less adaptability and flexibility.

The most likely explanation of increases in trunk variability during walking on grass with the cognitive task is that the combination of conditions (uneven surface and cognitive task) caused the highest demands on processing capacity which triggered the interference effect in the young adults that led to increased gait variability in terms of maintaining gait performance. Walking on an irregular surface requires more effort to control the movement, but at the same time effort is focused on the cognitive task, therefore the postural adjustment and anticipation might be less effective and the gait variability increases as a consequence.

### 4.5. Effect of Surfaces on Gait Complexity

Gait complexity determines complex fluctuations assessed in gait patterns which never precisely repeat themselves and which are responsible for the flexible adaptations to daily stresses placed on the human body during walking [1]. Despite growing interest, little is known about gait complexity behavior, especially in young adults and in the everyday environment. We observed that gait complexity in young adults remained unchanged during walking on various surfaces. This supported the above mentioned that even if young adult´s gait variability was higher, their gait pattern was still “stable”, due to the flexible adaptations and independent of external conditions. Tamburini et al. (2018) [65] came to the same conclusion when observing gait in indoor and outdoor conditions—environment affected the variability indexes, not the stability indexes (sample entropy).

### 4.6. Effect of Tasks on Gait Complexity

Although dual-task walking examination is popular and new tasks are constantly monitored, their observation in an outdoor environment is rather rare, especially if we focus on nonlinear gait assessment. Based on our results, the secondary task influenced gait complexity in young adults, when the complexity of trunk acceleration in the V direction tended to rise during performing cognitive tasks and to decrease during performing manual tasks.

That means that cognitive load resulted in a higher automaticity of the movement and less attention was involved in movement control—gait performance was more automatic and subcortical. Traditionally, higher complexity also indicated a higher adaptive system. A visuospatial task and listening to music during walking also led to increased SampEn values in young adults [34,35]. In contrast to our results, the increases were assessed in SampEn from trunk acceleration in the ML and AP directions [34].

In contrast, a manual load resulted in a lower automaticity of the movement. Indeed, holding and carrying a cup changed the posture, restricted the natural movement coordination—alternated upper limbs swing, and occupied attention, as spillage of water was unwanted and not allowed. In these conditions, the system was less adaptive and degrees of freedom were reduced. Magnani et al. (2017) [35] also reported decreased SampEn values in young adults during unilateral mobile phone handling with calling or texting compared to single-task walking.

### 4.7. Limitations

Our study also has several limitations. Firstly, we did not control the cognitive performance during the cognitive dual task. The main reason is associated with our aim to assess natural behavior during walking. We did not want to alter the gait performance due to stress, shame or fear arising from performance evaluation. In addition, we observed young adults, whose cognitive performance was usually intact (the number of mistakes is small) and the participants had different walking and also counting speeds (so there would be difference in the number of subtracting numbers/in the quantity of subtracting numbers). So, a higher number of mistakes in counting would not be necessarily associated with the changes in allocation of attention or with cognitive overload. Therefore, in our study the cognitive task was used only for cognitive simulation, when we walk and simultaneously solve a problem. Finally, we requested participants to do both tasks as well as possible.

Further, the findings are not fully comparable to everyday walking, because we asked participants to go ahead. In the real world, it is not usual to walk only straight on a flat path, we have to change direction and also manage the path’s inclines and descents.

## 5. Conclusions

In conclusion, we observed these patterns in motor controlled behavior. Irregular surfaces, as representative external constraints, provoke increases in gait variability. This statement supports the idea that higher variability is produced when the control system explores, in this case, the external environment and increases in variability represent rather a strategy which serves to find the most advantageous movement performance (solution) and allows flexible adaptation. It is important to note that physiological variability has its limits, although we are still unable to define them.

Conversely, tasks, as representative internal constraints, provoke mostly decreases in variability. Undoubtedly, tasks put higher demands on the motor control system. To maintain a successful performance, the motor control system slightly froze the exploring strategy and restricted the degrees of freedom, due to focusing effort on task fulfilment. Subsequently, motor performance is more automatic and subcortical in the case of cognitive load when there are no restrictions of body segments. In the case of manual load, when the natural movements of body are altered (carrying things), the gait pattern evinces less automaticity and more regularity.

The presented findings confirming significant changes in gait pattern induced by various conditions (surfaces, dual-tasking) are important for further daily life gait assessment (data collection without supervision), where it is almost impossible to control these conditions. Therefore, researchers should interpret the results obtained from daily life gait with caution. The effect of various conditions could also be exploited in gait training, where it would help physicians to build a more variable and effective intervention. The findings could also help improve gait examination (more demanding conditions could help detect discrete changes in gait pattern) and subsequent evaluation.

## Figures and Tables

**Table 1:** Gait velocity, root mean square (RMS) and sample entropy (SampEn) values.

				ST		Cognitive DT		Manual DT		Effect of Task	Effect of Surface	Interaction Effect
Variable	Signal	Plane/Direction	Surface	Mean	SD	Mean	SD	Mean	SD	*p*-Value	*p*-Value	*p*-Value
Gait speed			Asphalt	1.54	0.18	1.51	0.20	1.48	0.17	>0.05	>0.05	>0.05
(m·s^−1^)			Cobblestones	1.53	0.17	1.52	0.17	1.52	0.18			
			Grass	1.55	0.19	1.53	0.18	1.50	0.18			
RMS	acceleration	V	Asphalt	0.316	0.086	0.307	0.078	0.285	0.071	<0.001	>0.05	>0.05
(g)			Cobblestones	0.322	0.077	0.319	0.062	0.308	0.072			
			Grass	0.332	0.074	0.344	0.083	0.315	0.077			
		ML	Asphalt	0.178	0.051	0.169	0.046	0.156	0.039	<0.001	0.006	>0.05
			Cobblestones	0.184	0.047	0.177	0.038	0.174	0.043			
			Grass	0.203	0.044	0.209	0.050	0.197	0.054			
		AP	Asphalt	0.235	0.053	0.226	0.051	0.211	0.044	0.004	0.002	>0.05
			Cobblestones	0.243	0.055	0.232	0.037	0.233	0.049			
			Grass	0.268	0.053	0.275	0.064	0.258	0.058			
SampEn	acceleration	V	Asphalt	0.562	0.121	0.569	0.101	0.542	0.112	0.006	>0.05	>0.05
			Cobblestones	0.565	0.107	0.576	0.098	0.567	0.120			
			Grass	0.572	0.114	0.592	0.132	0.564	0.118			
		ML	Asphalt	0.817	0.118	0.838	0.129	0.832	0.112	>0.05	>0.05	>0.05
			Cobblestones	0.810	0.108	0.805	0.128	0.828	0.136			
			Grass	0.768	0.117	0.771	0.127	0.764	0.132			
		AP	Asphalt	0.525	0.132	0.515	0.100	0.520	0.094	>0.05	>0.05	>0.05
			Cobblestones	0.519	0.110	0.503	0.110	0.527	0.143			
			Grass	0.458	0.097	0.499	0.155	0.491	0.100			

RMS—root mean square; SampEn—sample entropy; ST—single-task; DT—dual-task; SD—standard deviation; V—vertical direction; ML—mediolateral direction; AP—anteroposterior direction.

## Data Availability

The data presented in this study are available on request from the corresponding author.
